# Resveratrol induces cell cycle arrest via a p53-independent pathway in A549 cells

**DOI:** 10.3892/mmr.2014.3100

**Published:** 2014-12-16

**Authors:** LONG YUAN, YONGRONG ZHANG, JUAN XIA, BIN LIU, QINGYU ZHANG, JIE LIU, LIMING LUO, ZHOU PENG, ZEQING SONG, RUNZHI ZHU

**Affiliations:** 1 Department of Respiratory Medicine, Affiliated Hospital of Guangdong Medical College, Zhanjiang, Guangdong 524001, P.R. China; 2 Laboratory of Hepatobiliary Surgery, Zhanjiang Key Laboratory of Hepatobiliary Diseases, Affiliated Hospital of Guangdong Medical College, Zhanjiang, Guangdong 524001, P.R. China; 3 Department of Respiratory Medicine, The Fourth Hospital of Zhanjiang, Zhanjiang, Guangdong 524001, P.R. China

**Keywords:** resveratrol, p53, cell cycle, apoptosis

## Abstract

Resveratrol, a non-flavone polyphenol compound, has a chemopreventive and chemotherapeutic effect against the progression of multiple types of cancer, including lung cancer. However, the molecular mechanism underlying the effects of resveratrol on cancer remain to be elucidated. In the present study, using an MTT assay, it was demonstrated that resveratrol inhibited cell proliferation in a concentration- and time-dependent manner. In addition, morphological features were observed in the A549, human lung cancer cell line, which included cell shrinkage, cells became distorted, certain cells became rounded and there was a concentration-dependent increase in the number of sloughed cells. Cell cycle analysis revealed that resveratrol may induce cell cycle arrest in the G_0_/G_1_ phase by downregulating the expression levels of cyclin D1, cyclin-dependent kinase (CDK)4 and CDK6, and upregulating the expression levels of the CDK inhibitors, p21 and p27. The immunofluorescence and western blot analysis results revealed that resveratrol upregulated the nuclear expression of p53 in A549 cells. Further studies have demonstrated that p53 downregulation did not contribute to the G_0_/G_1_ cell cycle arrest induced by resveratrol. In addition, resveratrol had no effect on the expression of p21, through use of the p53 inhibitor, pifithrin-α. The present study may offer a scientific basis for the further in-depth evaluation of resveratrol in the association of p53 and cell cycle arrest.

## Introduction

**Figure 1:**
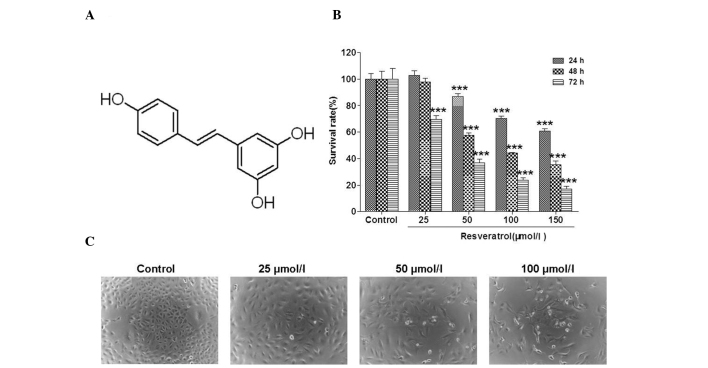
Resveratrol inhibited the proliferation of A549 non-small cell lung cancer cells. (A) Structure of resveratrol. (B) A549 cells were treated with 25, 50 and 100 μmol/l resveratrol for 24, 48 and 72 h separately. Cell viability was determined using an MTT assay and the survival rate (%) was calculated. Results are representative of three independent experiments. (C) Morphological changes induced by resveratrol were observed using an Olympus microscope. Images were captured at ×100 magnification. Values are expressed as the mean ± standard deviation (n=3),^***^P<0.001, vs. the control at each time point.

Resveratrol, a non-flavone polyphenol compound with a stilbene structure (Fig. 1A), is highly enriched in several dietary and pharmaceutical sources, including grapes, peanuts, berries, red wine and *Polygonum cuspidatum* (1,2). Previous studies have revealed that it has unique, beneficial effects on human health, such as cardiovascular protection, lifespan prolongation, anti-inflammatory effects, microcirculation improvement and regulation of lipid metabolism. In addition, a previous study documented that resveratrol has a chemopreventive and chemotherapeutic effect against the progression of various types of cancer, including prostate, breast, liver, skin and lung cancer (3).

Cancer is a disease characterized by loss of control over cellular growth, which evolves, in part by over-riding the regulation of cellular proliferation (4). The progress of the cell cycle in cancer cells is regulated by three protein families: Cyclins, cyclin-dependent kinases (CDKs) and CDK inhibitors (CDKIs). CDKs are critical regulators of the cell cycle machinery, which, when activated, provide a means to progress the cell cycle from one phase to the next (5). However, multiple changes occur in cancer cells, including cyclin amplification, CDK or substrate mutation, as well as inactivation of inhibitors. This results in abnormal CDK activity, amplification of positive growth signals, mutation of checkpoint and surveillance genes, as well as dysregulation of programmed cell death or apoptotic processes, inducing the selective growth advantage of cancer cells (6). Therefore, identifying agents that may induce cell cycle arrest has become a goal of cancer therapy, including small molecule inhibitors and gene therapy.

p53, the tumor suppressor gene product, is a key component in the regulation of cell cycle progression, which is activated in response to a wide spectrum of stresses and damage (7). A study demonstrated that p53 negatively regulates cell cycle progression in response to different cellular stresses (8). Commonly, when activated by genotoxic stress, p53 may directly regulate the p21^WAF1/CIP1/SDI1^ gene, which encodes a universal inhibitor of CDKs, to inhibit the cell cycle progression (9).

The present study aimed to investigate the anti-cancer effects of resveratrol on the A549 lung cancer cell line in order to confirm the function of the p53-independent pathway in resveratrol-induced cell cycle arrest in A549 cells.

## Materials and methods

### Reagents and antibodies

Resveratrol, purchased from Sigma-Aldrich (St. Louis, MO, USA), was dissolved at a concentration of 50 mmol/l in dimethysulfoxide (DMSO; MP Biomedicals, LLP, Santa Ana, CA, USA) stored at −20°C and diluted with Dulbecco’s modified Eagle’s medium (DMEM; Gibco-BRL, Carlsbad, CA, USA) to the desired working concentrations. The final concentration of DMSO did not exceed 0.4% (v/v) throughout the study. Pifithrin-α was purchased from Sigma-Aldrich and diluted to a final concentration of 5 mg/ml. DAPI was obtained from Beyotime Institute of Biotechnology (Haimen, China).

The primary monoclonal human anti-rabbit antibodies against cyclin D1 (#2926), CDK4 (#2906), CDK6 (#3136), p21 (#2947), p27 (#2552), p53 (#9282) and GAPDH (#2118) were all obtained from Cell Signaling Technology, Inc. (Danvers, MA, USA). The horseradish peroxidase-conjugated anti-rabbit (E030220) and anti-mouse IgG secondary antibody was purchased from EarthOx (San Francisco, CA, USA) and the Alexa Fluor 488 labeled anti-rabbit IgG secondary antibody was obtained from Beyotime Institute of Biotechnology.

### Cell culture

A549 cells were obtained from the Cancer Cell Repository (Shanghai Cell Bank, Shanghai, China). Cells were maintained in DMEM supplemented with 10% (v/v) heat-inactivated fetal bovine serum (Gibco-BRL) and antibiotics (100 U/ml penicillin and 100 U/ml streptomycin; Hyclone Laboratories, Inc., Logan, UT, USA) at 37°C in a humidified atmosphere of 5% CO_2_.

### Cell proliferation assay

The cells were plated at a density of 5×10^4^ cells/ml in 96-well microtiter plates. Different concentrations of resveratrol (25, 50, 100 and 150 μm/l) were used to treat cells for 24, 48 and 72 h. A total of 20 μl MTT solution (5 mg/ml; Sigma-Aldrich) was transferred to each well. Plates were incubated for 4 h at 37°C. Following incubation, supernatants were removed and 100 μl DMSO was added to ensure total solubility of formazan crystals. After 15 min of agitation, the absorbance was measured at 570 nm using a plate reader (Perkin-Elmer Inc., Waltham, MA, USA). The survival ratio was calculated using the following equation: Survival ratio (%) = OD _treated_/OD _control_ × 100.

### Cell cycle analysis

For cell cycle analysis, the cells were plated at a density of 5×10^4^ cells/ml in 6-well microtiter plates for 24 h adherence and treated with different concentrations of resveratrol or without for 48 h. Following treatment, the cells were harvested by trypsinization and fixed in ice-cold 70% methanol overnight at −20°C. The cells were subsequently centrifuged at 300 × g for 5 min and incubated with a propidium iodide (PI) working solution (100 μg/ml PI and 100 μg/ml RNaseA) for 30 min at 37°C. Cell cycle distribution was analyzed using a FACScan flow cytometer (FACSCalibur; BD Biosciences, Franklin Lakes, NJ, USA).

### Western blot analysis

The A549 cells were treated with various concentrations of resveratrol (25, 50 and 100 μm/l) for 48 h and lysed with radioimmunoprecipitation assay lysis buffer (Beyotime Institute of Biotechnology, Jiangsu, China). The cell lysates were centrifuged at 13,000 × gfor 10 min and the supernatant was collected. Protein concentration was determined using the Bradford protein assay (10). Total protein (20 μl) was separated on 10–15% sodium-dodecyl sulfate polyacrylamide gel electrophoresis and transferred onto a 0.45 μm polyvinylidene difluoride membrane (Millipore, Billerica, MA, USA) in a buffer containing 25 mmol/l Tris-HCl (pH 8.3), 192 mmol/l glycine and 20% methanol. The membranes were then blocked with 5% fat-free dry milk in Tris-buffered saline and Tween-20 containing 0.05% Tween-20 for 2 h and then incubated with primary antibodies overnight at 4°C. Subsequently, treatment with the appropriate horseradish peroxidase conjugated secondary antibody was performed at a dilution of 1:2,000. The immunoreactive bands were detected using the enhanced chemiluminescence method.

### Immunofluorescence staining

Cells were incubated for 48 h with or without resveratrol. All sloughed and attached cells were harvested, fixed with ice-cold 4% paraformaldehyde (Beyotime Institute of Biotechnology) for 10 min and washed with ice-cold phosphate-buffered saline (PBS). Cells were then permeabilized with 0.3% Triton X-100 (Beyotime Institute of Biotechnology), washed with ice-cold PBS (Jinuo, Hangzhou, China), stained with antibodies against P53 and subsequently incubated with Alexa Fluor secondary antibodies (Invitrogen Life Technologies, Carlsbad, CA, USA). Cell nuclei were stained with DAPI (Beyotime Institute of Biotechnology) and the cells were observed using a fluorescence microscope (TCS SP5II, Germany) with peak excitation wavelengths at 570 nm and 460 nm.

### Statistical analysis

Values are presented as the mean ± standard deviation of at least three independent experiments. Data were evaluated using the Student’s t-test and GraphPad Prism version 5 (GraphPad Software, Inc., La Jolla, CA, USA). P<0.05, P<0.01 or P<0.001 were considered to indicate statistically significant differences between values.

## Results

### Resveratrol inhibits cell proliferation in A549 cells

To examine the antitumor effects of resveratrol, A549 cells were treated with different concentrations of resveratrol for 24, 48 and 72 h and then cell viability was determined using an MTT assay. As shown in Fig. 1B, resveratrol inhibited the cell viability of A549 cells in a concentration- and time-dependent manner. As the microscopy images in Fig. 1C show, following exposure to 25, 50 or 100 μmol/l resveratrol for 48 h, the A549 cells exhibited morphological features, including shrinkage and distortion; in addition, certain cells became rounded, while the number of adherent cells decreased significantly, which was consistent with the cell proliferation assay.

### Resveratrol induces cell cycle arrest in G_0_/G_1_ phase

**Figure 2:**
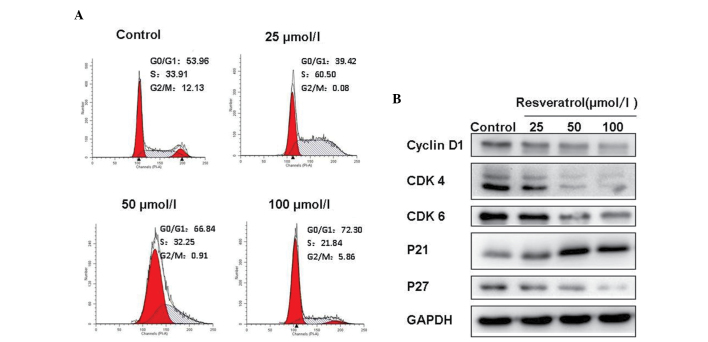
Resveratrol induced cell cycle arrest at G_0_/G_1_ and S phase in A549 cells. (A) Cell cycle analysis. A549 cells were treated with 25, 50 and 100 μmol/l resveratrol for 48 h, and then cells were harvested and stained with propidium iodide for 30 min at 37°C. Cells were then subjected to flow cytometric analysis to determine the cell distribution at each phase of cell cycle. (B) Western blot analysis. Cells treated with resveratrol were lysed for western blot analysis with antibodies against G_0_/G_1_-associated proteins, including cyclin D1, CDK 4 and 6, and CDK inhibitors p21 and p27. GAPDH was used as a reference gene. CDK, cyclin-dependent kinase.

To demonstrate the possible mechanism of resveratrol-induced cell growth inhibition in A549 cells, cell cycle progression was analyzed. A549 cells were treated with 25, 50 or 100 μmol/l resveratrol for 48 h and then the cell cycle phase was analyzed using flow cytometry. As shown in Fig. 2A, upon treatment with 25 μmol/l resveratrol, the S phase distribution increased between 33.91 and 60.50%, but with the increase in the concentration of reveratrol, the percentage of cells in S phase was decreased. While the G_0_/G_1_ phase distribution was increased between 53.96, 66.84 and 72.30% when treated with 25, 50 and 100 μmol/l resveratrol respectively, this was accompanied by a cell cycle G_0_/G_1_ phase distribution increase and S phase distribution decrease.

### Effects of resveratrol on proteins involved in G_0_/G_1_ phase arrest

In view of the progression of the cell cycle in vertebrate cells, cells prepare for S phase during the G_1_ phase, which is triggered by regulation of cyclin D1, CDK4 and CDK6 (11,12). The formation of cyclin D1-CDK4/6 complexes regulate the synthesis of DNA to prepare for cell division, while the CKIs, including p21 and p27 have a negative regulatory role in this process (13,14). The western blot analysis (Fig. 2B) revealed that resveratrol treatment reduced the protein levels of cyclin D1, CDK4 and CDK6 significantly, and resulted in upregulation of the proteins levels of p21 and p27 in a concentration-dependent manner.

### Resveratrol induces cell cycle arrest, however not via p53

**Figure 3:**
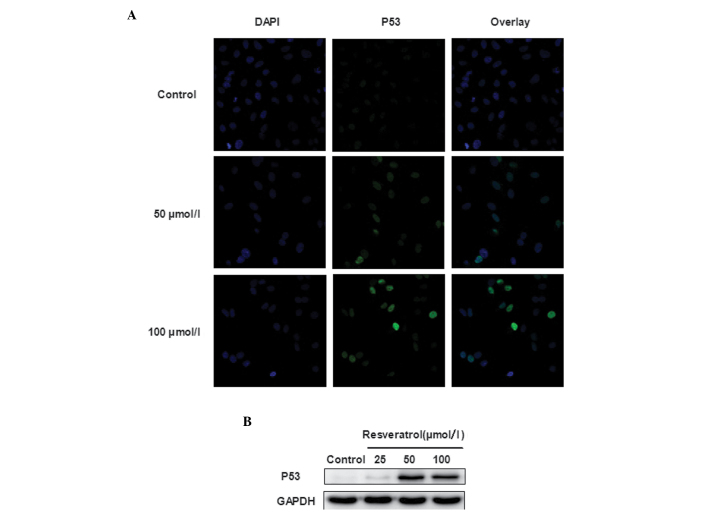
Resveratrol upregulated nuclear p53 accumulation in A549 cells. (A) A549 cells were treated with 25, 50 and 100 μmol/l resveratrol for 48 h and DAPI localization of nuclei (left) and the cellular localization of p53 (middle) are revealed by immunofluorescence as well as the overlay (right). Results are representative of three independent experiments. Representative images of immunocytofluorescence demonstrate the subcellular localization and the expression of p53. DAPI provided staining of nuclear DNA. The cells were monitored by confocal fluorescence microscopy. (B) Western blot analysis. Cells treated with resveratrol were lysed for subjection to western blot analysis with an antibody against p53. GAPDH was used as a reference gene.

**Figure 4:**
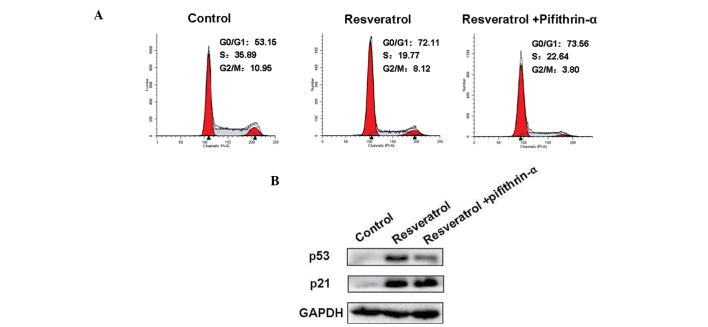
Resveratrol induced p53-independent cell cycle arrest. (A) Cell cycle analysis. A549 cells were treated with 100 μmol/l resveratrol with or without pifithrin-α pre-treatment, then the cells were harvested and stained with propidium iodide for 30 min at 37°C. The cell cycle distribution was detected by flow cytometric analysis. (B) Western blot analysis. Cells treated were lysed for subjection to western blot analysis with antibodies against p53 and p21. GAPDH was used as a reference gene.

p53 is a regulator of the cell cycle and the inactivation of p53 has an important role in tumor formation (15). However, it remains to be determined whether p53 is involved in cell cycle arrest induced by resveratrol. As shown in Fig. 3A and B, it was demonstrated that resveratrol upregulated the protein level of p53 significantly. Resveratrol treatment resulted in nuclear accumulation of p53, inducing apoptosis in A549 cells. To confirm the role of p53 in resveratrol-induced cell cycle arrest, pifithrin-α, a p53 inhibitor was used to pre-treat A549 cells. The results demonstrated that p53 downregulation did not induce cell cycle arrest (Fig. 4A) or regulate p21 (Fig. 4B) in A549 cells.

## Discussion

Resveratrol is currently being evaluated as a potential cancer chemopreventive compound (16). In the present study, the chemopreventive/therapeutic potential of resveratrol was evaluated in the A549 lung cancer cell line, and its underlying mechanism was examined. Consistent with previous observations, it was identified that resveratrol may inhibit cell proliferation through the induction of cell cycle arrest in A549 cells in a time- and concentration-dependent manner (17). However, an important observation of the present study is that resveratrol induced cell cycle arrest in the G_0_/G_1_ phase, this was accompanied by p53 upregulation, which may contribute to the antitumor effect of resveratrol. However, the cell cycle arrest induced by resveratrol was not reversed following treatment with an inhibitor of p53, pifithrin-α.

The potent cytotoxic action of resveratrol was demonstrated by a time- and concentration-dependent decrease in cell viability of A549 cells, which was concomitant with an increase in the percentage of cells that underwent cell cycle arrest at the G_0_/G_1_ phase. Different classes of cyclins and their complex formation with CDKs control the different phases of cell cycle progression (18). G_0_/G_1_ phase provides the materials for mitosis, and DNA replication occurs during S phase, which is regulated by cyclin D1, CDK4 and CDK6. In the present study, flow cytometric analysis revealed that resveratrol induced A549 cells to arrest in the G_0_/G_1_ phase and it was associated with a decrease in the protein level of cyclin D1, CDK4 and CDK6. CDK-cyclin complexes, the motors of the cell cycle, are regulated by two families of CKIs, the CIP/KIP family (p21^WAF/CIP^, p27^KIP1^ and p57^KIP2^) and the INK4 family (p15INK4b, p16INK4a, p18INK4c and p19INK4d) (19). p21^WAF1/CIP1^, a member of the CIP/KIP family, binds to CDK/cyclin complexes and prevents kinase activation, subsequently halting the cell cycle in the G_1_ phase. In response to cell cycle arrest induced by antitumor agents, it was demonstrated that the cell cycle may be regulated through either p53-dependent or p53-independent pathways (20,21). In the present study, p21^WAF1/CIP1^ was upregulated by resveratrol in A549 cells accompanied with a significant increase in the protein level of p53. The present results provided a possible mechanism, through which resveratrol upregulated the expression level of p21^WAF1/CIP1^ and thereby led to G_0_/G_1_ phase arrest through a p53-dependent pathway. However, further investigation revealed that the inhibition of p53 had no effect on resveratrol-induced cell cycle arrest and the expression of P21^WAF1/CIP1^.

In conclusion, the present study may offer a scientific basis for the further in-depth evaluation of the tumor suppressor, resveratrol. Resveratrol inhibited cell proliferation in a concentration- and time-dependent manner in A549 cells by inducing cell cycle arrest at the G_0_/G_1_ phase via p21 upregulation, accompanied by p53 activation. However, the resveratrol-induced p21-mediated G_0_/G_1_ arrest was not dependent on the p53 pathway, which indicated that p53 may not exhibit effects on cell-cycle arrest-induced cell growth in A549 cells.
